# IL-10 Induction from Implants Delivering Pancreatic Islets and Hyaluronan

**DOI:** 10.1155/2013/342479

**Published:** 2013-07-22

**Authors:** Paul L. Bollyky, Robert B. Vernon, Ben A. Falk, Anton Preisinger, Michel D. Gooden, Gerald T. Nepom, John A. Gebe

**Affiliations:** ^1^Division of Infectious Diseases and Geographic Medicine, Department of Medicine, Stanford University School of Medicine, Grant Building, 300 Pasteur Drive, Stanford, CA 94305-5107, USA; ^2^Benaroya Research Institute, 1201 Ninth Avenue, Seattle, WA 98101-2795, USA

## Abstract

Local induction of pro-tolerogenic cytokines, such as IL-10, is an appealing strategy to help facilitate transplantation of islets and other tissues. Here, we describe a pair of implantable devices that capitalize on our recent finding that hyaluronan (HA) promotes IL-10 production by activated T cells. The first device is an injectable hydrogel made of crosslinked HA and heparan sulfate loaded with anti-CD3/anti-CD28 antibodies and IL-2. T cells embedded within this hydrogel prior to polymerization go on to produce IL-10 *in vivo*. The second device is a bioengineered implant consisting of a polyvinyl alcohol sponge scaffold, supportive collagen hydrogel, and alginate spheres mediating sustained release of HA in fluid form. Pancreatic islets that expressed ovalbumin (OVA) antigen were implanted within this device for 14 days into immunodeficient mice that received OVA-specific DO.11.10 T cells and a subsequent immunization with OVA peptide. Splenocytes harvested from these mice produced IL-10 upon re-challenge with OVA or anti-CD3 antibodies. Both of these devices represent model systems that will be used, in future studies, to further evaluate IL-10 induction by HA, with the objective of improving the survival and function of transplanted islets in the setting of autoimmune (type 1) diabetes.

## 1. Introduction

 Interleukin 10 (IL-10) is a potent immunosuppressive cytokine made by regulatory T cells (Tregs) and other cell types [1–3]. IL-10 inhibits antigen-specific immune responses in part *via* suppression of activated macrophage and monocyte functions, which include cytokine synthesis and expression of class II MHC and costimulatory molecules such as IL-12 and CD80/CD86 [4].

IL-10 has important roles in transplant biology. Endogenous IL-10 production is correlated with transplant acceptance in multiple animal models and human tissues [5–8]. IL-10 has been evaluated as a treatment to improve the survival of engrafted islets, which has been accomplished by transfer of IL-10-producing Tregs [6], gene therapy [9, 10] or direct administration of IL-10 alone, or in conjunction with immunomodulatory drugs [11–13]. It is noteworthy that systemic IL-10 treatment has failed to support islet engraftment in mice in the setting of established autoimmunity [14] and may induce immune suppression. These results suggest that an alternative approach that provides a sustained, local presence of IL-10 at the graft site might be more effective at preventing islet rejection.

 We recently reported a role for the extracellular matrix (ECM) macromolecule *hyaluronan *(HA) in regulating IL-10 production by T cells. HA is a simple, long-chain glycosaminoglycan polymer made up of repeating disaccharides of N-acetyl glucosamine and glucuronic acid. HA is an important structural component of many tissues, but also has important roles in inflammation and tissue repair [15–18]. Short HA oligomers (<30 kDa) generated through tissue catabolism are typically proinflammatory [16–18]. Conversely, plate-bound HA or chemically crosslinked HA is anti-inflammatory and promotes IL-10 production by FoxP3(+) natural Tregs (nTregs) [19] and conventional T cells *in vitro* [20]. Induction of IL-10 in these systems was mediated by crosslinking of CD44, the primary receptor for HA [20]. We have proposed that plate-bound HA and HA hydrogels may function as biomimetics of HA-containing tissue matrices. However, the minimum size for HA-mediated CD44 crosslinking and IL-10 production by T cells is unknown. Additional support for a role for HA in IL-10 production is provided by observations of HA-induced upregulation of IL-10 by cultured synoviocytes [21] and elevated IL-10 levels in intestinal biopsies of mice given oral HA [22]. However, HA alone does not appear to promote IL-10 induction by T cells *in vitro*. Indeed, our data suggest that concomitant antigenic stimulation through the T cell receptor (TCR) complex is required for efficient IL-10 induction in the presence of HA.

 HA preparations are currently used as treatments for arthritis [23], atopic dermatitis [24], prevention of abdominal adhesions [25, 26] and are under evaluation as an experimental treatment for burns and wounds [27, 28]. In most of these preparations, HA is crosslinked to promote its stability and efficacy [29]. Crosslinking (as well as plate-binding or sustained release from alginate) may also limit the generation of pro-inflammatory HA fragments. Building upon these findings, we have evaluated whether HA has utility in promoting IL-10 production *in vivo*.

 Here, we describe and evaluate a pair of technologies that both provide antigenic stimulation in the context of HA. First, we have asked whether cells implanted within a crosslinked HA hydrogel that incorporates a supplemental complex to induce polyclonal TCR stimulation could enhance production of IL-10 *in vivo*. Second, we have developed a bioengineered implant capable of delivering an antigenic signal along with sustained release of HA in fluid form. These technologies represent parallel strategies for delivering HA as a medium to promote IL-10 production *in vivo*, with the ultimate objective of inducing durable immune tolerance to transplanted islets in individuals with autoimmune diabetes. 

## 2. Materials and Methods

### 2.1. Transgenic Mice

C57BL/6 green fluorescent protein (GFP)-FoxP3 knock-in and RIPmOVA/Rag2^−/−^ mice were the kind gifts of Dr. A. Rudensky (Memorial Sloan-Kettering Cancer Center, New York, NY, USA) and Dr. Steve Ziegler (Benaroya Research Institute—BRI), respectively. DO11.10 mice were purchased from Taconic Farms. All mice were maintained in a specific pathogen-free, AAALAC-accredited facility at BRI, and all experiments were approved by the BRI Institutional Animal Care and Use Committee (IACUC), protocol approval number 10116.

### 2.2. Isolation of Leukocyte Populations

 Mouse lymphocyte populations were prepared as previously described [19]. In brief, for the *in vitro* experiments, CD4(+) cells were isolated using MACS kits (Miltenyi, Inc.), and the GFP-FoxP3(−) fraction was isolated from the CD4(+) population using a FACS Vantage cell-sorter (BD Biosciences). CD4(+)/GFP-FoxP3(−) T cells were used to ensure that any IL-10 production we measured would be from conventional T cells, rather than from activated GFP-FoxP3(+) nTregs. Cells were cultured in Opti-MEM (Invitrogen) serum-free media supplemented with 100 *μ*g/mL penicillin and 100 U/mL streptomycin (P/S). Where specifically noted, cells were cultured in complete media consisting of Dulbecco's Modified Eagle's Medium (DMEM)-10 (Invitrogen) supplemented with 10% fetal bovine serum (FBS) (Hyclone), P/S, 50 *μ*M *β*-mercaptoethanol, 2 mM glutamine, and 1 mM Na pyruvate (Invitrogen). 

### 2.3. *In Vitro* T Cell Activation Using Plate-Bound Antibodies and HA

 Cell culture plates (96-well) were coated with 0.5 *μ*g/mL of anti-CD3 antibody (145-2C11, BD Biosciences), washed, and then subsequently coated with either 0.2 mg/mL bovine serum albumin (BSA)- conjugated HA (1.5 × 10^6^ Da) HA (Genzyme) or 10% BSA. CD4(+)/GFP-FoxP3(−) T cells (2 × 10^5^ per well) were cultured for 96 hours on these plates, followed by collection of the culture supernatants for analysis. Measurement of cytokines in the cell culture supernatants was performed using enzyme-linked immunosorbent assays (ELISAs) or cytometric bead assays (BD Biosciences). 

### 2.4. *In Vitro* T Cell Activation Using HA Hydrogels

 Hydrogels were made from thiolated constituents (HA, heparin sulfate [HS], and collagen) crosslinked with polyethylene glycol S-S diacrylate (PEGSSDA). These reagents are available as a kit (Extracel-HP, Glycosan/Biotime) and were used per the manufacturer's instructions. Of note, our understanding from communications with the manufacturer is that HA of >1 × 10^6^ Da is used in the kits. Prior to addition of the crosslinker, the mixture was supplemented with 10 *μ*g/mL of streptavidin (Sigma Aldrich), 10 *μ*g/mL each of biotinylated anti-CD3 and anti-CD28 antibodies (145-2C11, 37.51, BD Biosciences), and 20 IU/mL of IL-2. Hydrogels of this formulation are referred to here as *“supplemented HA hydrogels.”* For *in vitro *cell culture experiments, 2 × 10^5^ CD4(+)/GFP-FoxP3(−) T cells were layered on top of 25 *μ*L volumes of the hydrogel. After 96 hours of culture, cells and culture supernates were collected for analysis. To control the collagen constituent of the HA hydrogels, a set of hydrogels lacking HA/HS was made by replacing the thiolated HA/HS with an equivalent volume of thiolated collagen. These controls are referred to as *“supplemented collagen hydrogels.”*

### 2.5. Implantation of T Cells and HA

 3 × 10^6^ CD4(+)/GFP-FoxP3(–) T cells were dispersed in supplemented HA hydrogels of 300 *μ*L volume prior to crosslinking with PEGSSDA, which was initiated 30 min prior to intraperitoneal injection into mice. For these studies *in vivo*, the supplemented HA hydrogels incorporated 360 IU/mL of IL-2. Four days after injection of the supplemented HA hydrogels, the mice were sacrificed and lymphoid tissues were harvested. Residual hydrogel material in the peritoneal cavity was also removed and dissolved by mild reduction of the PEGSSDA (per the manufacturer's instructions) in order to retrieve cells for analysis. Intracellular staining of these cells for IL-10 and subsequent flow cytometry assays utilized antibodies and equipment as previously described [19].

### 2.6. Isolation of Islets

 Islets were isolated as described previously [30]. Briefly, C57Bl/6 mice of 12–24 weeks age were anesthetized with 2,2,2-tribromoethanol in phosphate-buffered saline (PBS). The descending aorta of each anesthetized mouse was transected, the bile duct clamped at its distal (intestinal) end, and a 30-gauge needle was used to inflate each pancreas through the common bile duct with 4 mL of 4°C *Islet Medium *comprised of RPMI 1640 containing 1.0 g NaHCO_3_, 10% FBS (Atlanta Biologicals, cat. number S12450H), 1 mM Na-pyruvate, and P/S. The Islet Medium was supplemented with 0.8 mg/mL of collagenase P (Roche, cat. number 11-249-002-001) and filtered at 0.22 *μ*m prior to injection. Subsequently, 2-3 excised pancreata were placed in separate 50 mL conical centrifuge tubes and incubated in 5 mL of Islet Medium for 13 min at 37°C. The medium was then decanted, fresh 4°C Islet Medium was added, and the tubes were shaken vigorously to disrupt the pancreata. The tissue suspensions were filtered through a 30-mesh metal screen to remove large debris, the filtrates were pelleted by centrifugation, and the pellets resuspended in 4°C Islet Medium. The resuspended material was centrifuged through Histopaque 1077 to isolate the islets, which were washed, resuspended in Islet Medium, and placed in a tissue culture (TC) incubator. After all pancreata were processed, the isolated islets were hand picked, cultured overnight, and picked again the next day before being placed in bioengineered implants. Average yields were 100–150 islets per mouse.

### 2.7. Fabrication of Bioengineered Implants (BIs)

#### 2.7.1. Polyvinyl Alcohol (PVA) Scaffolds

Biopsy punches (Sklar Instruments) were used to cut 10 mm diameter disks from 2 mm thick sheets of PVA sponge (Type CF90, 500 *μ*m average pore size with no surfactant treatment—a generous gift from Merocel/Medtronic, Inc.). Subsequently, each disk was through-punched with a single central hole of 2 mm diameter and six peripheral holes of 1.5 mm diameter, using correspondingly sized biopsy punches (Acuderm, Inc.). The punched disks were washed 5 × 10 min on a rocker in 50 mL centrifuge tubes filled with 40 mL of sterile distilled water, then air-dried on Whatman filter paper, transferred to 60 mm dishes, sterilized by gamma irradiation, and stored until needed for BI assembly.

#### 2.7.2. Type I Collagen Solution

One volume of a stock solution of rat tail native type I collagen in dilute (0.02 N) acetic acid (BD Biosciences) was combined with 1/9 volume of 10-strength NaHCO_3_-saturated Medium 199 (Invitrogen) and sufficient DMEM and normal mouse serum (NMS) to yield a working solution containing 2.5 mg/mL collagen and 10% NMS [30]. The working solution was prepared just prior to assembly of the BIs and kept on ice until needed.

#### 2.7.3. Alginate Spheres

An aqueous stock solution of 4% alginate (Sigma-Aldrich, cat. number A0682), filtered at 0.45 *μ*m, was used for preparation of spheres for sustained release of vascular endothelial growth factor (VEGF) and HA. Spheres incorporating human recombinant VEGF_165_ (Peprotech, cat. number 100-20) were prepared as described previously [30]. Briefly, a mixture of 2% alginate and 5 ng/*μ*L VEGF was formed into 10 *μ*L (2.2 mm diameter) spheres using a gravity-drop method, crosslinked into a hydrogel for 15 min in 0.1 M CaCl_2_, washed 2 × 2 min in 0.15 M NaCl/25 mM HEPES/2 mM CaCl_2_, pH 7.2 (saline/HEPES/Ca), transferred to serum-free DMEM/P/S, and kept in a tissue culture incubator until needed for BI assembly.

Fabrication of HA spheres was similar to that of the VEGF constructs, with replacement of the VEGF with 50 *μ*g of 120 kDa HA (Genzyme). HA of this size (approximately 317 disaccharide units) was chosen to facilitate a complete delivery of HA from the spheres within a 2 week experimental time period.

#### 2.7.4. Assembly of BIs

Dry PVA sponge scaffolds were allowed to swell for 5 min in sterile DMEM/P/S. Subsequently, a single, freshly prepared alginate sphere containing VEGF and five spheres containing HA were gently pressed into the 6 peripheral holes of each expanded scaffold. The scaffolds were then blotted on sterile Whatman filter paper, transferred to 60 mm plastic cell culture dishes lined with UV-sterilized Parafilm M, and flooded with 60 *μ*L of type I collagen working solution containing suspended islets. The PVA sponges absorbed the collagen solution, with the majority of the islets entering the 2 mm diameter central hole of the scaffold. Subsequently, the dishes were covered with dish tops (lined with moist filter paper) and placed in a tissue culture incubator for 30 min to polymerize the collagen into a hydrogel. The completed BIs were placed in DMEM/10% NMS/P/S in 24-well cell culture plates and kept in a tissue culture incubator prior to implantation in mice.

#### 2.7.5. Measurement of Release of HA from Alginate Spheres *In Vitro*

To measure the kinetics of release of HA from alginate hydrogels *in vitro*, spheres containing 2% alginate and 2.5 *μ*g of fluorescein isothiocyanate (FITC)-conjugated 120 kDa HA or 1.5 × 10^6^ Da HA were prepared as described previously. The spheres were placed in 96-well cell culture plates (one sphere per well) that had each of the wells filled with 200 *μ*L of Dulbecco's Ca/Mg PBS (DPBS Ca/Mg, Invitrogen). The plates were placed in a tissue culture incubator, and 100 *μ*L volumes of the media were removed from each well at specific time points (up to 14 days) and analyzed by fluorescence spectrophotometry to quantify released FITC-HA, using a standard curve of fluorescence *versus* known concentration of FITC-HA. Following removal of the medium at each time point, the residual medium in each well was discarded, and each well was refilled with 200 *μ*L of fresh medium.

To determine the percentage of HA retained in alginate spheres during their fabrication, freshly prepared spheres containing 2.5 *μ*g of FITC-HA were dissolved in PBS/100 mM ethylenediaminetetraacetic acid (EDTA), and the resultant solution was analyzed by fluorescence spectrophotometry to quantify total FITC-HA per sphere.

#### 2.7.6. Implantation of BIs *In Vivo*

BIs were implanted into mesenteric pockets of RIPmOVA/Rag2^−/−^ mice (one BI per mouse) using previously described protocols [30], followed by injection of the mice 24 hours later with 1 × 10^5^ purified OVA-specific CD4(+) DO11.10 T cells.

### 2.8. Statistical Analyses

 Statistical comparison of samples was made using Student's *t*-test. 

## 3. Results

### 3.1. Supplemented HA Hydrogels Promote IL-10 Production *In Vitro*

We previously demonstrated that plate-bound HA together with an antigenic signal promotes IL-10 production by CD4(+)/GFP-FoxP3(−) T cells. This led us to ask whether we could develop this finding into a tool for use in promoting IL-10 production* in vivo*.

**Figure 1:**
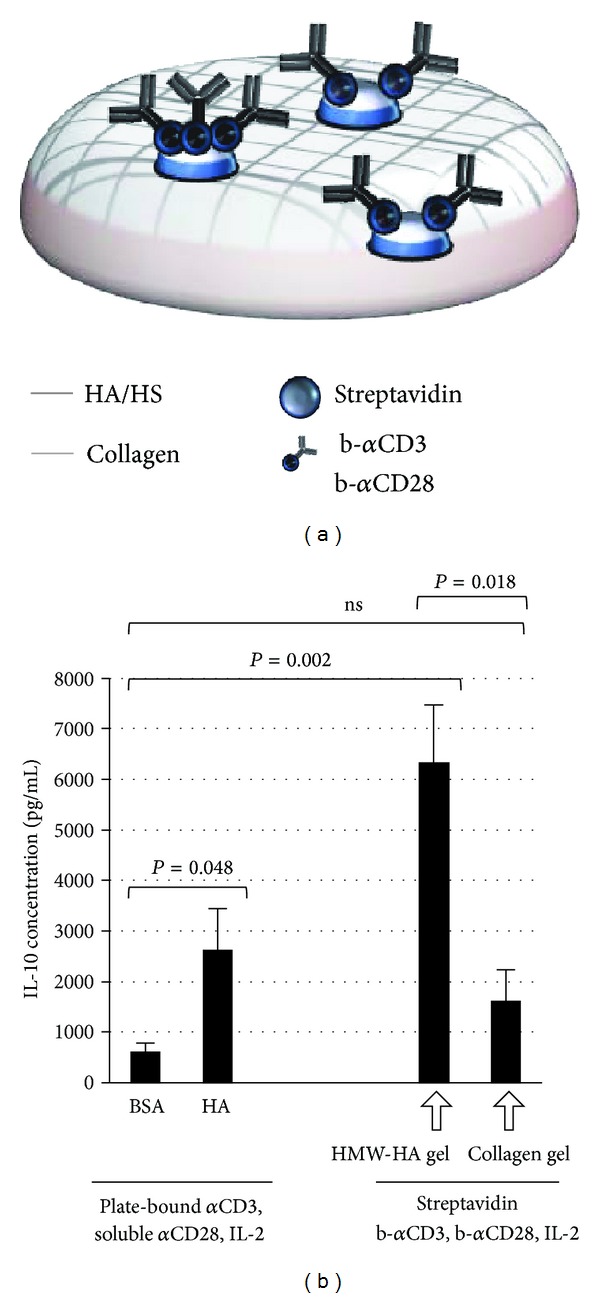
Induction of IL-10 by supplemented HA hydrogels *in vitro*. (a) Schematic for the design of a hydrogel capable of delivering a TCR stimulus in association with an HA signal. The “supplemented HA hydrogel” incorporates a crosslinked matrix of thiolated high molecular weight (HMW)-HA, HS, and collagen to which we have added streptavidin and biotinylated anti-CD3 and anti-CD28 antibodies (b-*α*CD3, b-*α*CD28) to deliver an activating signal through the TCR. (b) Concentration of IL-10 in supernates taken from T cell cultures 96 hours after either plate-based (left side of graph) or hydrogel-based (right side of graph) activation (cells were cultured on top of the hydrogels for the latter experiments). Supplemented collagen hydrogels that lacked HA were used as a negative control. *n* = 5 independent experiments.

 To this end, we modified a HA-based hydrogel to deliver a polyclonal antigenic stimulus through addition of streptavidin, biotinylated anti-CD3/CD28 antibodies, and IL-2. A schematic of this hydrogel design is shown in Figure 1(a). We have previously shown that a similar form of supplemented HA hydrogel is an efficient way to elicit IL-10 production from T cells *in vitro *[20].

We found that CD4(+)/GFP-FoxP3(–) T cells exposed to the supplemented HA hydrogels produced IL-10 at significantly higher levels than did corresponding T cells activated with anti-CD3/CD28 antibodies and IL-2 on cell culture plates (Figure 1(b)). This was the case whether the cells were cultured on top of the gels (as shown) or embedded within the gels (data not shown). Omission of either streptavidin or anti-CD3 antibody from the gel mixture likewise abrogated IL-10 production (data not shown), indicating that CD3 was required for the stimulus and suggesting that streptavidin was necessary to retain CD3 in the hydrogel lattice. Streptavidin, biotinylated anti-CD3/CD28 antibodies, and IL-2 incorporated into a hydrogel lacking HA (supplemented collagen hydrogel) did not significantly increase IL-10 production over plate-bound activation by these agents (Figure 1(b)), which demonstrated the potentiating influence of HA on IL-10 production. The unique capability of HA to stimulate IL-10 production by T cells is underscored by the observation that hydrogels made from other types of ECM, including basement membrane components (Matrigel) and fibrin, are not stimulatory *in vitro* [20].

### 3.2. Supplemented HA Hydrogels Promote IL-10 Production *In Vivo*

**Figure 2:**
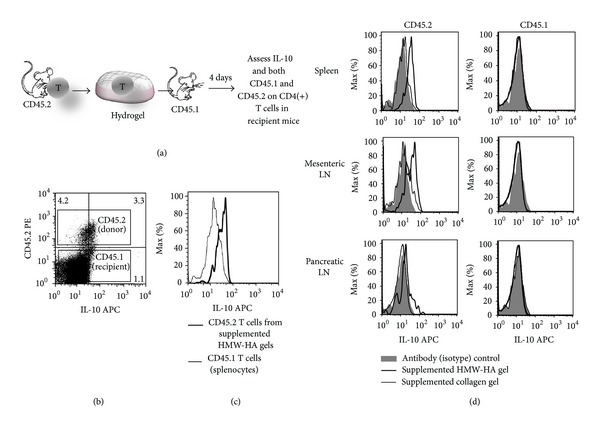
Supplemented HA hydrogels induce IL-10 *in vivo. *(a) Diagram of the experiment. Supplemented HA hydrogels, or control collagen hydrogels lacking HA, were populated with 3 × 10^6^ CD4(+)/GFP-FoxP3(–)/CD45.2 donor cells and injected into the peritoneal cavities of CD45.1 recipient mice. Four days after implantation, lymphoid tissues were harvested, processed, and stained for intracellular IL-10 and CD markers. (b) Gating indicates relative IL-10 expression by CD45.2 donor and CD45.1 host T cells. (c) IL-10 expression by CD45.2 donor T cells harvested from HA hydrogel residue removed from the peritoneal cavity is substantially greater than that of CD45.1 host T cells from the spleen. (d) IL-10 staining of CD3(+)/CD4(+) T cells harvested from the spleen and mesenteric/pancreatic lymph nodes (LN). The donor T cells from mice that received the supplemented HA hydrogels expressed higher levels of IL-10 than corresponding donor T cells from mice that received the control supplemented collagen hydrogels. Host T cells from these two groups of mice did not express IL-10 above levels of the nonspecific antibody (isotype) controls. In (c), and (d), data are representative of three experiments each.

To evaluate whether supplemented HA hydrogels could be used to induce IL-10 production *in vivo*, the gels were populated with 3 × 10^6^ CD4(+)/GFP-FoxP3(–) T cells from CD45.2 mice and injected into the peritoneal cavities of CD45.1 mice. By use of the CD45.1 and CD45.2 allelic markers, the donor and recipient cell populations could be distinguished. A schematic of this transfer protocol is shown in Figure 2(a). Four days after implantation, spleens and lymph nodes were harvested, processed, the released cells stained for intracellular IL-10 and CD markers, and gating performed to distinguish donor T cells from host T cells (Figure 2(b)). As controls, analogous supplemented collagen hydrogels lacking HA were populated with cells and injected into a designated set of mice. 

 After 4 days, a substantial volume of residual HA hydrogel was found within the peritoneal cavities of the treated mice; however, the control collagen hydrogels had dissolved. In separate experiments, we found that after 7 days no implanted HA hydrogels were identifiable, indicating that extensive catabolism of the hydrogels takes place *in vivo*. 

The cells within the HA hydrogel residue 4 days after implantation were primarily CD45.2(+) and expressed IL-10 at a high level relative to host T cells from the spleen (Figure 2(c)). These cells remained FoxP3(−) (data not shown), consistent with our previous report that HA does not induce FoxP3 expression [19]. Cell isolates from the spleens and lymph nodes of the transplanted mice contained CD45.2(+) donor T cells (Figure 2(d)), which indicated that the T cells embedded in the hydrogels had migrated into lymphoid tissues. Donor T cells that migrated from the supplemented HA hydrogels expressed higher levels of IL-10 than the corresponding donor T cells that migrated from the control collagen hydrogels. Host T cells from these two groups of mice did not express IL-10 above levels of the nonspecific antibody controls (Figure 2(d)). These data indicate that HA hydrogels providing endogenous TCR stimuli can be used as platforms to induce IL-10 production *in vivo*. 

 While supplemented HA hydrogels are a novel system for inducing implantable T cell populations that produce IL-10, we sought to devise an implantable platform that would elicit IL-10 production from endogenous T cells in an antigen-specific manner. To this end, we adapted a novel *bioengineered implant (BI)* we had developed from an earlier study to combine the antigenic stimulus with sustained release of HA within the same construct.

### 3.3. Sustained Release of HA from BIs Induces IL-10 Production *In Vivo*

We recently reported on the development of the BI as a model system to explore improved approaches for islet transplantation [30]. The BI, sized for mesenteric or subcutaneous implantation in mice, consists of a disk-shaped PVA sponge infused with a type I collagen hydrogel that contains dispersed donor islets. To promote islet vascularization, the BI incorporates a spherical alginate construct for delivery of VEGF. Previously, we used syngeneic mice to demonstrate that BIs containing 450–500 islets and 20 ng of VEGF could reverse streptozotocin (STZ)-induced diabetes in 100% of recipients [30]. Notably, none of these mice required exogenous insulin therapy once the BIs began to fully regulate levels of blood glucose. Moreover, the transplanted mice responded to glucose challenge in a near-normal manner.

Induction of pro-tolerogenic cytokines, such as IL-10, is an appealing strategy to help facilitate transplantation of islets. Here, we have adapted our BI device to evaluate the capacity of HA in fluid form (i.e., HA not crosslinked to form a hydrogel) to elicit IL-10 production in an autoimmune setting. To test this model, we loaded the BI with islets expressing the OVA antigen, transferred in OVA-specific T cells, immunized the recipient mice with OVA, and asked whether these cells expressed IL-10 in an OVA or TCR-specific manner.

**Figure 3:**
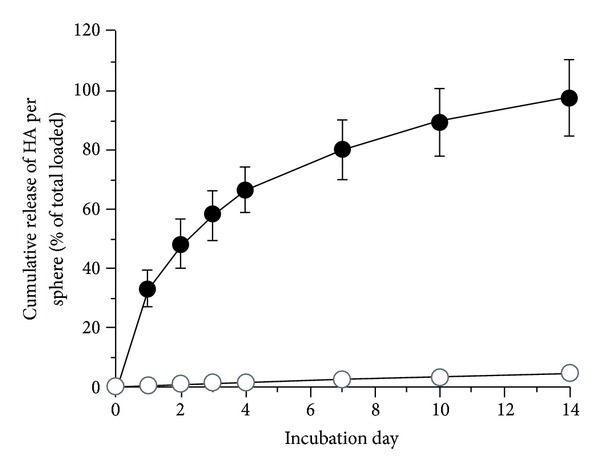
Kinetics of release of HA from alginate *in vitro*. Alginate spheres (2% alginate/10 *μ*L volume/2.2 mm diameter) incorporating 2.5 *μ*g of FITC-labeled 120 kDa HA (closed circles) or FITC-labeled 1.5 × 10^6^ Da HA (open circles) were cultured at 37°C in DPBS Ca/Mg, and the media collected at specific time points for measurement of released HA. Essentially all (98%) of the 120 kDa HA was released over 14 days, whereas less than 5% of the 1.5 × 10^6^ Da HA was released during this time period. Error bars = standard deviations (error bars for the 1.5 × 10^6^ Da group are within the diameter of the open circles). *n* = 10 spheres for each group.

**Figure 4:**
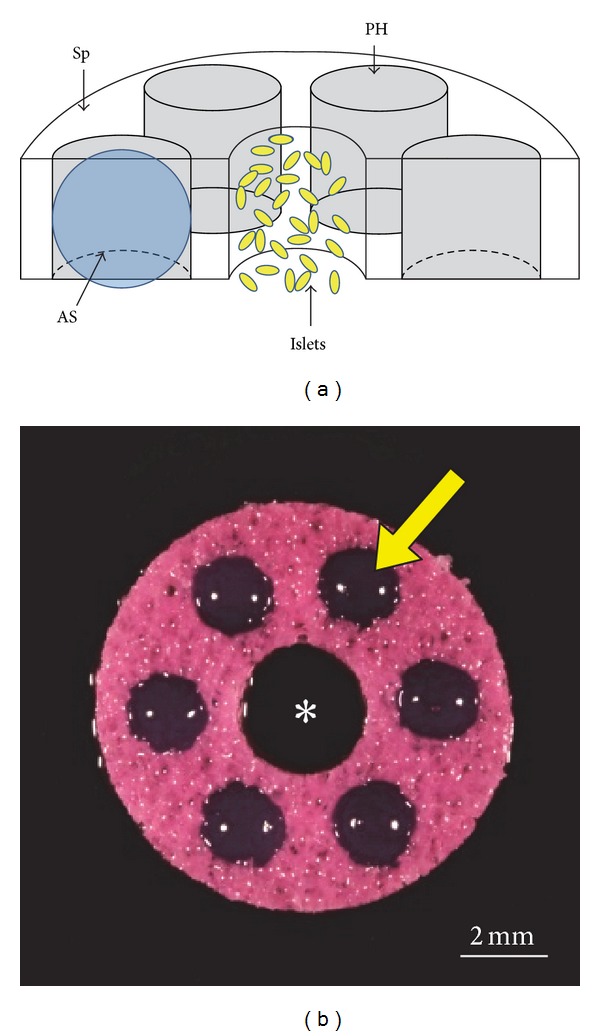
BI designed for evaluation of immune modulation by HA *in vivo*. (a) Cut-away diagram of the BI. A disk-shaped PVA sponge (Sp) scaffold provides mechanical support. Alginate spheres (AS-blue) occupy the six peripheral holes (PH) of the sponge. A central hole in the sponge contains islets (yellow) suspended in a type 1 collagen hydrogel (not shown). The collagen hydrogel also infuses the sponge. For clarity, the pores of the sponge are not depicted. (b) A PVA sponge scaffold (stained pink from culture medium) is oriented to show the empty central hole (asterisk) and the six peripheral holes, which each contain an alginate sphere (e.g., arrow). The scaffold is 10 mm in diameter × 2 mm thick.

**Figure 5:**
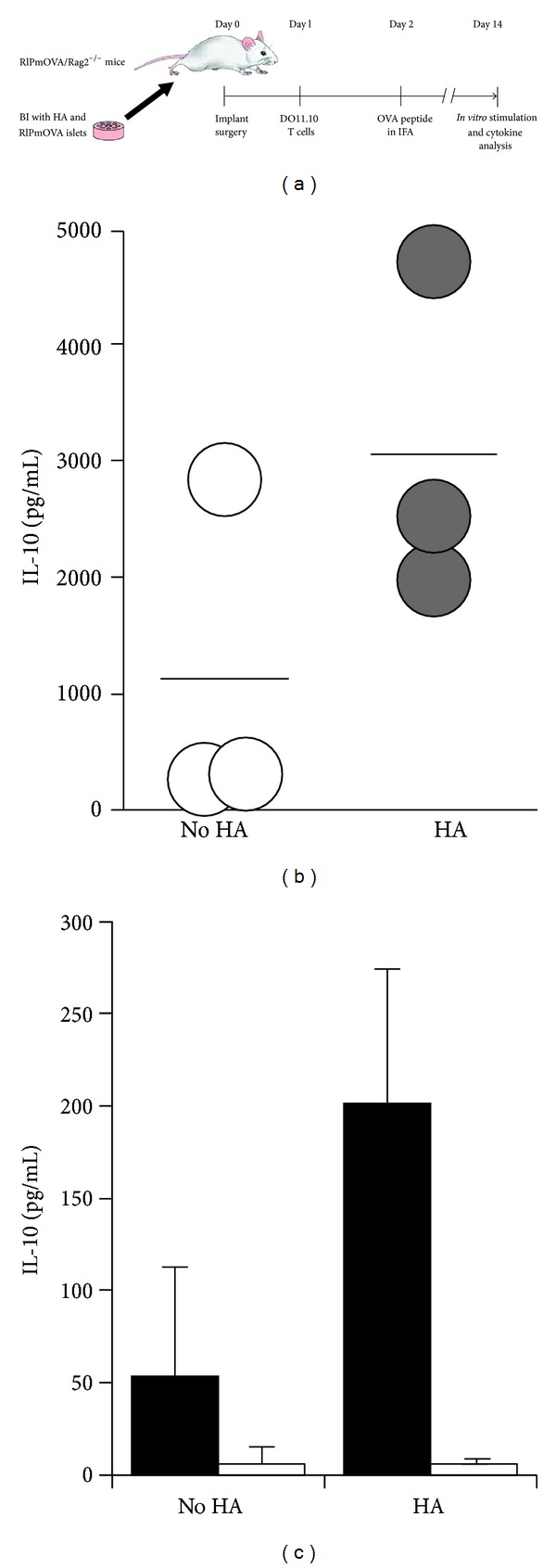
Local release of fluid HA from BIs generates IL-10 producing T cells *in vivo*. (a) Diagram of the experiment. BIs incorporating RIPmOVA islets and alginate spheres loaded with HA or saline (controls) were implanted in RIPmOVA /Rag2^−/−^ mice, followed by injection of OVA-specific DO11.10 T cells on day 1 and 100 *μ*g of OVA peptide in incomplete Freund's adjuvant (IFA) on day 2. On day 14, splenocytes were harvested and assayed for IL-10 production in response to antigenic stimulation *in vitro*. (b) IL-10 production by splenocytes from recipient animals in response to stimulation with anti-CD3/CD28 antibodies. (c) IL-10 production in response to OVA peptide (black bars) or no peptide (white bars). In (b) and (c), cells were stimulated for 96 hours, and culture supernates in contact with the stimulated cells for 72 hours were assayed by ELISA. Data are representative of 2 experiments totaling 5 mice in each group.

 We first evaluated the kinetics of release of HA from 2 mm diameter, 2% alginate spheres under physiological conditions *in vitro* (Figure 3). We found that release of 1.5 × 10^6^ Da HA was linear, but relatively slow—less than 5% was released after 14 days. In contrast, the release of 120 kDa HA was much more rapid—essentially 100% was released within 14 days, which was a useful time frame in which to analyze post-transplantation immune responses. We found that over 60% (63.7% ± 6.1%) of the 120 kDa HA loaded into each sphere was retained by the alginate after crosslinking with calcium. The BIs we designed for our experiments *in vivo* (Figure 4) incorporated a single alginate sphere containing 20 ng of VEGF and 5 spheres that contained a total of 160 *μ*g of 120 kDa HA (the total is derived from a value of 32 *μ*g of HA per sphere, based on 64% retention of the 50 *μ*g of HA present in each sphere prior to calcium crosslinking). A set of control BIs incorporated one VEGF sphere and 5 spheres loaded with saline in place of the HA. The central hole of the BI was infused with a type I collagen hydrogel containing islets from RIPmOVA mice, which express chicken ovalbumin driven by the rat insulin promoter (RIP). BIs of this design were implanted into mesenteric pockets of RIPmOVA/Rag2^−/−^ mice (one BI per mouse), followed by injection of the mice 24 hours later with 1 × 10^5^ purified OVA-specific CD4(+) DO11.10 T cells. Forty-eight hours after implantation, each mouse was immunized with 100 *μ*g of OVA peptide (aa 323–339). On day 14 after implantation, splenocytes were isolated from the mice and assayed for IL-10 production *in vitro* after 96 hours of stimulation with either anti-CD3/anti-CD28 antibodies or antigen-specific OVA peptide (Figure 5(a)). In this assay, the splenocytes from the mice treated with HA produced more IL-10 than the splenocytes from the control mice that were not exposed to HA. This differential response was observed when the splenocytes were given either a nonspecific stimulus with CD3/CD28 (Figure 5(b)) or a specific stimulus with OVA peptide (Figure 5(c)). Unfortunately, these data do not allow us to discern which cells are the source of IL-10 in this assay and specifically whether the cells in question are FoxP3(+) Tregs or FoxP3(−) conventional T cells.

## 4. Discussion 

 We demonstrate, using two separate model systems, that delivery of HA together with antigenic signals promotes the production of IL-10 *in vivo*. Our data suggest a potential clinical application for HA-mediated induction of IL-10-producing T cells using injectable hydrogels. HA hydrogel platforms are in development for a variety of applications, including drug delivery and wound dressings, and are noted for their biocompatibility [31, 32]. In the present study, we have shown that augmentation of HA hydrogels with a complex of biomolecules that provide TCR stimulation in addition to the HA signal can deliver the requisite cues for IL-10 induction, both *in vitro* and *in vivo*.

In treatments of diabetic patients that involve transplantation of islets, controlling rejection is typically accomplished by systemic immunosuppressive compounds. Dosing of these compounds is a difficult balance—levels must be low enough to permit a reasonable degree of protective immunity against pathogenic organisms, but high enough to effectively suppress allo- and autoimmune activity. In the case of simultaneous pancreas-kidney (SPK) transplants, some current immunosuppression regimens are inadequate to control autoimmunity [33, 34]. Moreover, no matter what the dose, systemic immunosuppression can be accompanied by a variety of undesirable side-effects on tissue and organ systems that are not directly associated with the transplant. In light of the problems associated with systemic treatments, an alternative approach would be to confine the delivery of immunotherapy to the implant itself. In this way, immunomodulatory compounds could be delivered at relatively high concentrations, but within the limited volume of the implant, thereby minimizing side-effects on tissues and organs outside the zone of delivery. To this end, the BI described here includes a mechanically-supportive scaffold and ECM hydrogel that concentrates the islets in a small volume, and a sustained-release component for local delivery of immunomodulatory compounds.

In the present study, we have adapted our BI to release HA in fluid form. Rather than using HA of 1–1.5 × 10^6^ Da that is typically incorporated into HA hydrogels, we used HA with a 10-fold lower MW (120 kDa) to provide release kinetics that were optimal for the 14-day duration of our experiments *in vivo*. Of note, we observed that this shorter HA could induce an IL-10 response from host mice. To our knowledge, this observation is the first demonstration of IL-10 production by HA of this weight class. It is possible that the 120 kDa HA is crosslinked into higher MW forms after its release into tissue from alginate, which could be accomplished by HA-binding molecules such as inter-alpha-trypsin inhibitor (I*α*I) and/or tumor necrosis factor-stimulated gene-6 protein (TSG-6) which are present at sites of inflammation and which are known to crosslink HA into macromolecular assemblies [35–37]. Such crosslinking could result not only in a functional increase in the MW of HA, but also promote the retention of HA in the fibrovascular tissue that invades the BI [30] and in the tissues immediately surrounding the implant. Our future studies will continue to use BIs as platforms to evaluate the effectiveness of HA and other specific ECM and cytokine environments on islet survival and reversal of diabetes in the setting of autoimmunity.

## Abbreviations

HA: hyaluronan;
BRI: Benaroya Research Institute;
BI: bioengineered implant;
GFP: green fluorescent protein;
IL-2, IL-10: interleukin-2, -10;
HS: heparan sulfate;
NMS: normal mouse serum;
TCR: T cell receptor complex;
Tregs: regulatory T cells;
VEGF: vascular endothelial growth factor.
